# Efficiency of Lift Production in Flapping and Gliding Flight of Swifts

**DOI:** 10.1371/journal.pone.0090170

**Published:** 2014-02-28

**Authors:** Per Henningsson, Anders Hedenström, Richard J. Bomphrey

**Affiliations:** 1 Department of Biology, Lund University, Lund, Sweden; 2 Structure & Motion Lab, The Royal Veterinary College, University of London, Hatfield, Hertfordshire, United Kingdom; Scientific Institute Foundation Santa Lucia, Italy

## Abstract

Many flying animals use both flapping and gliding flight as part of their routine behaviour. These two kinematic patterns impose conflicting requirements on wing design for aerodynamic efficiency and, in the absence of extreme morphing, wings cannot be optimised for both flight modes. In gliding flight, the wing experiences uniform incident flow and the optimal shape is a high aspect ratio wing with an elliptical planform. In flapping flight, on the other hand, the wing tip travels faster than the root, creating a spanwise velocity gradient. To compensate, the optimal wing shape should taper towards the tip (reducing the local chord) and/or twist from root to tip (reducing local angle of attack). We hypothesised that, if a bird is limited in its ability to morph its wings and adapt its wing shape to suit both flight modes, then a preference towards flapping flight optimization will be expected since this is the most energetically demanding flight mode. We tested this by studying a well-known flap-gliding species, the common swift, by measuring the wakes generated by two birds, one in gliding and one in flapping flight in a wind tunnel. We calculated span efficiency, the efficiency of lift production, and found that the flapping swift had consistently higher span efficiency than the gliding swift. This supports our hypothesis and suggests that even though swifts have been shown previously to increase their lift-to-drag ratio substantially when gliding, the wing morphology is tuned to be more aerodynamically efficient in generating lift during flapping. Since body drag can be assumed to be similar for both flapping and gliding, it follows that the higher total drag in flapping flight compared with gliding flight is primarily a consequence of an increase in wing profile drag due to the flapping motion, exceeding the reduction in induced drag.

## Introduction

Any flying device, whether it is a bat, a bird, an insect or an airplane, generates lift with a measureable efficiency. An ideal wing that generates lift in the most efficient way does so by deflecting the oncoming airflow uniformly across the span to achieve an elliptic lift distribution; this configuration generates the smallest amount of induced drag [1]. By measuring the shape of this distribution and quantifying how large the deviation from uniformity is, it is possible to calculate the efficiency of lift generation [2]–[8]. Flying animals, unlike aircraft, generate both lift and thrust by flapping their wings, but many animals also use gliding flight as a large proportion of their routine behaviour. The challenge for animals that flap and glide is that both of these flight modes need to be performed using the same set of wings, yet the design optimum for a flapping wing is different from one intended solely for gliding. When flapping, a velocity gradient is created across the span because the tip of the wing travels faster than the root. To compensate for this difference in velocity and thereby maintain a uniform downwash, either the wing chord needs to reduce towards the tip (i.e. a tapering wing planform) or the local angle of attack needs to be reduce (i.e. a twisted wing) – or both at the same time. In the gliding case, however, where the wings are held stationary, the optimal wing shape is elliptic with no twist and—to reduce the relative effect of the wing tip vortices—the ellipse should have high aspect ratio.

The animal can potentially adapt to these two very different tasks, flapping and gliding, by morphing its wings (e.g. [9]), but only within the limitations of its anatomy. The animal is faced with a trade-off – either its wings need to have a shape that is a perfect compromise, resulting in equally sub-optimal performance in both flight modes, or the shape will be biased towards better performance in one of the flight modes. If we consider the latter case, is it better to be more efficient when flapping or gliding? We hypothesised that higher efficiency when flapping would be of greater advantage than when gliding because energy expenditure through muscle recruitment is far greater during this flight mode [10]. The null-hypothesis is consequently that there is no difference between gliding and flapping span efficiencies. We tested this by measuring the downwash profiles of flying common swifts (*Apus apus* L.) using high speed particle image velocimetry (PIV) in a wind tunnel. The swifts spend almost their entire life on the wing, landing almost only during breeding [11], and the typical flight manner of swifts is flap-gliding, making it a suitable species for this study. In a flap-gliding flight mode the bird flaps its wings to gain altitude and/or speed and then switch to gliding for some period of time before resuming flapping. The swifts typically alternate between flapping and gliding flight, flapping at about 60% of the time in free flight [12].

## Materials and Methods

### Study animals

Two juvenile common swifts were captured on two separate occasions in their nests in the early morning on their estimated fledging day. The birds were kept in a lidless plastic box (0.5×0.4 m) with an artificial nest bowl, which is an appropriate housing for juvenile swifts since it resembles the nest environment. They were hand fed every other hour from morning to evening with a mixture of minced insects, vitamins and water using a syringe. Morphological details of the two birds are shown in Table 1, which also shows that the two birds were similar in body mass, wing shape and body frontal area.

**Table 1:** Morphological details of the two birds used in the experiments.

Parameter	Flapping	Gliding
Mass (kg)	0.042	0.042
Wingspan (m)	0.38	0.39
Wing area (m^2^)	0.014	0.016
Mean wing chord (m)	0.037	0.041
Wing aspect ratio	10.3	9.8
Body frontal area (m^2^)	0.0011	0.0011
Second moment of wing area	1.09×10^−4^	1.52×10^−4^
Third moment of wing area	1.34×10^−5^	1.99×10^−5^

The use of the swifts in experiments and the capture of them were approved by the Ethics Committee at Lund University (Permit number: M-204-06) and the license for catching the birds for experiments was issued by the Swedish Environmental Protection Agency (permit number: 412-4636-03). All efforts were made to care for the birds and to minimize stress for them while in captivity and during wind tunnel flights. Both birds were released into the wild in good condition after finishing the experiments.

### Wind tunnel

The Lund University wind tunnel is a low-turbulence, closed-circuit tunnel designed for experiments with live animals. The bird used for the flapping flight experiments was flown in the wind tunnel at three speeds: 5.9, 7.8 and 10.0 m/s and the bird used in the gliding flight experiments was flown at five speeds: 7.0, 8.0, 9.0, 10.0 and 11.0 m/s. The measurements of the gliding bird were done with the wind tunnel tilted to the best glide angle for the bird at each speed (the glide angle where the bird performs its highest lift-to-drag ratio; [13]. Further details on the two sets of experiments can be found in [13] and [15]. The test section is 1.22 m wide and 1.08 m high. Air speed across 97 per cent of the test section is within ±1.3 per cent of the mean and the baseline turbulence is approximately 0.03 per cent of the mean [16].

### Particle Image Velocimetry

The tunnel was seeded with a thin mist (particle size 1 µm) using an aerosol generator. The mist was illuminated by a pulsed 50 mJ laser (Litron LPY732, Nd:YAG, 532 nm) at a repetition rate of 200 Hz. The laser beam was spread by a cylindrical lens into a sheet, approximately 2 mm thick, directed from above transverse to the flow. Flow field areas of approximately 0.2×0.2 m (sufficient to record half the span of the swifts) were captured by two CMOS-sensor cameras (High-SpeedStar3: 1024×1024 pixels) connected to frame grabber PCI boards on a host pc and synchronised with the laser and each other using a high-speed controller. The cameras were equipped with 60 mm lenses (AF Micro Nikkor 60 mm f/2.8D) set to aperture 2.8. The system was controlled using DaVis 7.2.2 software package (LaVision, Göttingen, DE). Cameras were calibrated using the calibration routine in DaVis and type 22 LaVision dual-plane calibration plate. The calibration was further refined using the self-calibration routine, which accounts for small misalignment between the calibration plate and the laser sheet at the time of calibration.

During experiments the system was triggered manually when the bird was flying steadily in the appropriate location, approximately 8–11 chord lengths upstream from the laser sheet. At this downstream distance there may have been an effect of wake deformation prior to measurement [17] although we expect this to be small due to the flight speed of the birds. Each recording lasted one second in the flapping experiments and half a second in the gliding experiments, resulting in 200 and 100 vector field measurements respectively. If the bird moved too close to the laser sheet during a measurement the light was immediately suspended for the safety of the bird. During recordings the bird was allowed to fly freely in the test section; only sequences containing steady flight were used for further analysis.

The PIV data were processed using DaVis 7.2.2. Raw images were filtered by subtracting a sliding minimum over 5 frames to remove disturbances in the images, such as streaks in the light or if the bird was visible in the background. After filtering, multi-pass stereo cross-correlation was performed at an interrogation window size of 32×32 pixels with 50% overlap. Vector fields were post-processed in two steps: *i*) vectors that showed a peak ratio <1.01 when dividing the highest correlation peak with the second highest correlation peak were deleted, and *ii*) vectors with a magnitude 2 times the neighbourhood root mean square (RMS) were deleted and recalculated if the magnitude was 3 times the neighbourhood RMS. Finally, empty spaces were filled up by interpolation, a 3×3 smoothing average was applied and freestream velocities, based on separate measurements of the freestream flow, were subtracted. All vector field sequences have been made available on the DRYAD repository as DaVis VC7-files (doi:10.5061/dryad.cn252). Note that only sections within these sequences that correspond to steady flight, where the complete semi wake span was captured, were used for analysis.

### Data analysis

The method used here to extract downwash distributions from the wake has been described in detail previously by [2] and [5], and therefore only the main features are described here. The position of the wingtip vortex core (of left or right wing depending on which semi-span was captured) and the position of the body centre in each vector field were manually digitised using a custom-written Matlab script (MathWorks, Inc., Natick, MA, USA). A linear transect was drawn between the centre position and the vortex core position and the velocity vectors closest along this line were extracted by the script. These vectors were considered representative of the induced flow distribution along the wing. In order to sample the full width of the wake, the transects were extended to also capture the velocity vectors outboard of the wingtip vortex core centres. The data were mirrored at the sagittal plane to create a full wake span. The centreline was easily located since either both tail vortices or both wing root vortices were visible in the frames – the location of the centreline was defined as the point between these two structures.

The velocity transects were used to calculate the instantaneous lift and span efficiency. Lift was calculated from the vertical component of the velocity vectors by integrating the elemental contribution to lift along the span according to
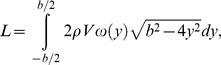
(1)where ρ is the density of the air, *V* is the freestream velocity, ω(*y*) is spanwise vertical velocity, *y* is spanwise location and *b* is the span of the wake.

The distribution of velocity vector components perpendicular to the transect were taken as the induced flow distribution created by the wings and span efficiency was calculated as
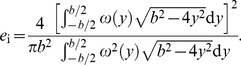
(2)


The induced flow distribution was approximated as an analytic function by fitting a cosine series with five harmonics to the data. For the flapping case, phase-averaged lift and span efficiency were calculated. Wingbeat average span efficiency was calculated for each sequence as the ratio between the total ideal induced power and the real (measured) induced power according to
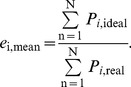
(3)


For the gliding case, arithmetic means of span efficiency and lift for each sequence were calculated. In several of the gliding flight sequences the wake of the tail was prominent. In order to assess the extent of the effect of the tail wake on span efficiency, a separate analysis with the tail wake removed from the velocity transects was performed. This was done by identifying the two tail vortices in the transects and replacing the velocities between them with the mean velocity. This way the variation due to the tail vortices was removed but the contribution to lift was retained.

Two different birds were used for the experiments – one for gliding and one for flapping flight. Swifts are notoriously difficult to keep in captivity and that in combination with the challenge of doing PIV experiments on freely flying birds made this experimental design necessary in order to be able to perform the study. The mass and morphology of the two birds were similar, but they naturally differed to some small degree (Table 1). To investigate the potential effect of these morphological differences we calculated the second and third moment of wing area of the two birds ([18]–[19]). These two parameters take into account the distribution of area along the span and are therefore considered a better measure of shape than more basic measures such as aspect ratio. The chord length at increments of 10 mm from base to tip of the wings were measured from top-down outline photographs and second and third moment of area were calculated as 
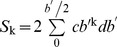
, where *k* = 2 and 3, *b*' is the wingspan, and *c* is the chord length at the different spanwise locations. The parameter values are presented in Table 1.

## Results

The average wingbeat frequency of the swift in flapping flight was 9.8, 9.9 and 8.9 Hz for the three speeds and with a sampling rate of 200 Hz that resulted in 20, 20 and 22 frames recorded per wingbeat. The criteria for a suitable sequence were strict: steady flight and both centreline and wingtip vortex of either left or right wing in view at all times. With a total measurement area of 0.2×0.2 m and a freely flying bird, this left very little margin since the wake semi-span of the swifts is just shy of 0.2 m. As a consequence, the total number of wingbeats analysed was limited to 5, 12 and 7 for the three speeds, respectively, but with a total number of vector fields of 484 for the flapping flight. The gliding flight analysis, following the same criteria for acceptance of sequences, included 119, 116, 162, 78 and 24 instantaneous measurements of span efficiency for the five speeds respectively.

**Figure 1:**
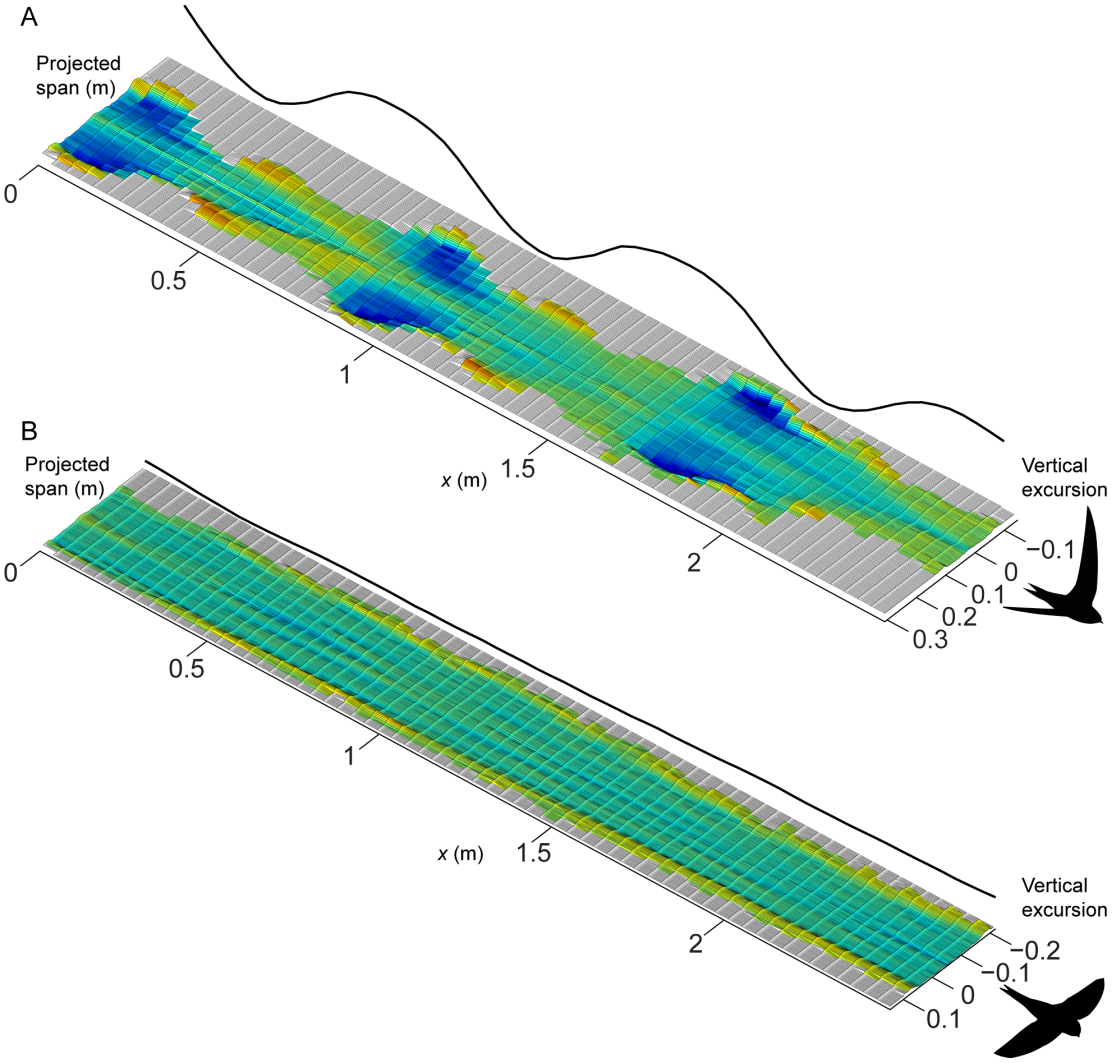
Examples of induced flow tracks behind the swifts at 8 m/s. Colour and relief both show magnitude of induced flow, with shades of blue showing downward velocities corresponding to positive lift and shades of red/yellow showing upward velocities corresponding to negative lift. Both panels show the same colour range and the solid line plotted on the far side of the graphs shows the vertical position of the wingtip vortex throughout the sequence; in the flapping case showing the flapping motion and in the gliding case indicating how steadily the bird was gliding. A) Three consecutive wingbeats behind the swift in flapping flight. Average *e*_i_ over the sequence was 0.67. B) An example of a gliding sequence cut to equal length as the flapping example. Average *e*_i_ was 0.56 for this sequence.

**Figure 2:**
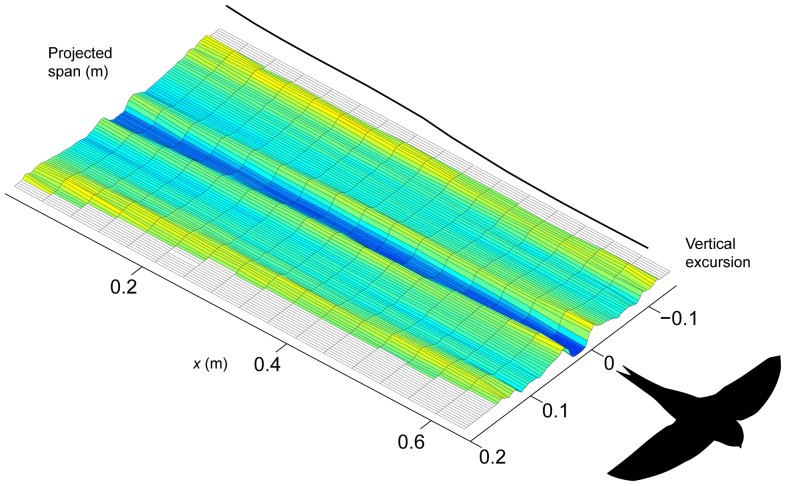
Example showing the influence by the tail in the induced flow track behind the gliding swift. This downwash resulted in an average *e*_i_ of 0.36.

Figure 1 shows examples of the induced flow behind the swifts at 7.8 m/s in flapping flight (Fig. 1A) and at 8.0 m/s in gliding flight (Fig. 1B). These illustrations are compilations of measured transects of induced velocities across the span of the flying birds with a displacement in between transects based on the flight speed of the swift so that the axis in direction of travel (*x*-axis) represents both time and space. The flapping flight plot shows how the induced flow is increased during the early part of the downstroke and reaches a peak just after mid downstroke. At supination the induced flow velocities decrease but remain into the early part of the upstroke. The remainder of the upstroke is largely inactive, generating almost no induced flow. The average span efficiency (eq. 3) over this particular sequence was *e*_i,mean_ = 0.67. The gliding flight plot shows a fairly invariable wake throughout this example sequence. The most prominent feature is perhaps the clear sections of upwash generated at the outer sections of the wingtip vortices. The average span efficiency (arithmetic mean of all instantaneous measurements in the sequence) was for this sequence *e*_i,mean_ = 0.56. At a few instances a weak trace caused by the action of the tail can be seen (e.g. at *x* = 0.5 to *x* = 1.0), but a clearer illustration of this is shown in Figure 2. In this sequence it is clear that the bird uses the tail to generate extra lift or for stabilisation (cf. [13], [14]). The investigation of the effect of the tail wake on span efficiency showed that, as expected, span efficiency was increased when the tail wake was removed, but it was only increased by on average 12% across all sequences.

**Figure 3:**
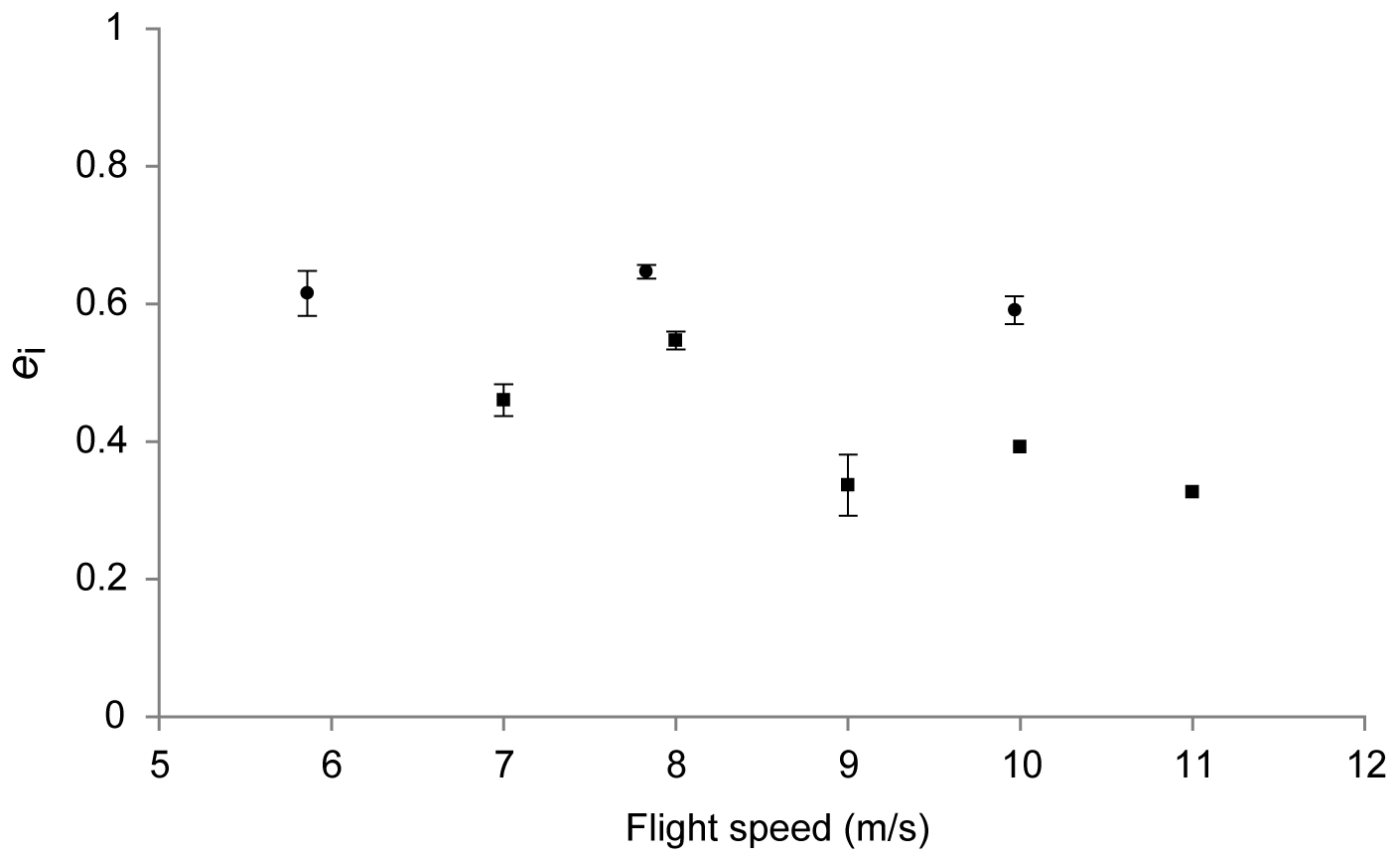
Average span efficiency across the range of flight speeds measured. Circles correspond to flapping flight and squares correspond to gliding flight. Error bars show standard error of the mean between sequences. Span efficiency is consistently higher in flapping flight than in gliding.

Average span efficiencies over the measured speed range in flapping and gliding flight are presented in Figure 3. Span efficiency was consistently higher in flapping than in gliding flight, and with this result we can reject the null hypothesis. The average span efficiency in flapping flight was 0.62 and the highest was 0.64 at 7.8 m/s. In gliding flight, the average was 0.41 and the highest was 0.55 at 8.0 m/s (two-samples t-test of all measurements across all speeds in flapping and gliding flight, respectively, gives *t* = 6.29. d.f. = 18, *p*<0.001). Average normalised lift (lift divided by weight of the bird) was 1.5±0.16 in flapping flight, and 1.2±0.09 in gliding flight, showing that even though lift was calculated from just the transects drawn through the vector fields, it was sufficient to capture the gross forces generated. The normalised lift for the same data calculated by measuring the circulation of the vortex structures in the wake gave on average 0.57±0.012 and 1.02±0.03 for flapping and gliding flight respectively [13], [15], so the force deficiency for flapping flight using that method is no longer present using the induced flow based calculation.

**Figure 4:**
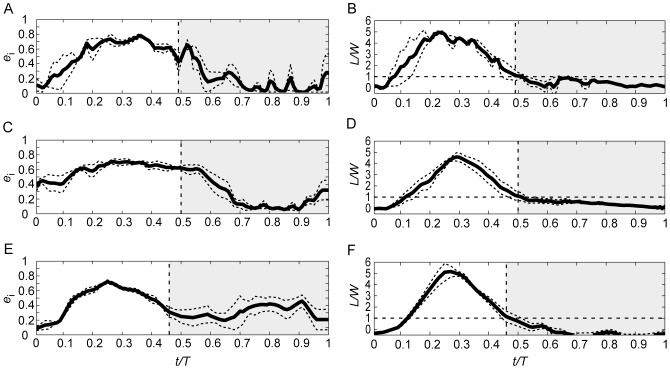
Phase-averaged span efficiency and normalised lift for the three speeds of flapping flight plotted over standardized wingbeat duration. Top row (A–B) shows span efficiency and lift at 5.9 m/s, middle row (C–D) for 7.8 m/s and bottom row (E–F) for 10.0 m/s. Solid curves show average and dashed curves show standard error of the mean. Vertical dashed lines mark the instance of supination. White areas correspond to the downstroke part of the wing stroke and grey-shaded areas correspond to upstroke.

The phase-averaged time history of span efficiency and normalised lift during flapping flight at the three speeds are shown is Figure 4. Consistent with the patterns shown in the induced flow track in Figure 1, very little lift is generated at pronation (also consistent, as expected, with the findings by [15] and [20]) but quickly builds up and, at t/T≈0.1, lift is already equal to the weight of the bird. Lift peaks at t/T≈0.3 at all three speeds, which is slightly after mid downstroke. In all three speeds lift decreases below weight support at the time of supination (t/T≈0.5), but some lift is still generated into the beginning of the upstroke, until t/T≈0.6, when lift is nearly zero. The remainder of the upstroke is close to inactive, generating only very small forces.

Span efficiency shows a slightly different pattern compared with lift; it rises in the beginning together with lift, but instead of a pronounced peak at around mid downstroke it stays more constant throughout the downstroke and into the beginning of the upstroke. When lift drops down close to zero, span efficiency also drops and stays low for the rest of the upstroke. At the highest speed, 10 m/s, an increase of span efficiency at the later stage of the upstroke is shown (Fig. 4E), but as can be seen in Figure 4F, this corresponds to a period of negative lift.

## Discussion

The swift in flapping flight had consistently higher span efficiency than the swift in gliding flight. This might be a counterintuitive result, since this species is renowned for its gliding behaviour. It has also been shown previously that swifts have much higher lift-to-drag ratio (*L*/*D*) in gliding than in flapping flight (*L*/*D* = 12.5 for gliding and 7.7 for flapping [13], [15]), and that they benefit from their flap-gliding flight mode because of this difference [21]. What is the mechanistic basis of this apparent paradox? It is important to point out here that span efficiency only relates to one type of drag, the induced drag (or drag due to lift), while parasite drag (the drag due to the body) and profile drag (the combined effect of skin friction and pressure drag of the wings) are excluded. It may be reasonable to assume that parasite drag stays constant irrespective of whether the swift is flapping or gliding and we have seen that induced drag is lower in flapping than in gliding because span efficiency is higher. That *L*/*D* (the result of all drag components) is superior in gliding flight than when flapping, despite the increase in induced drag when gliding, leaves wing profile drag as the main component responsible for the increase in the total drag. Thus, the increase in profile drag due to the flapping motion, which results in an increase in the average wing airspeed, exceeds the reduction in induced drag.

Normalised lift calculated using the downwash velocities from the transects resulted in values greater than one (1.5 for flapping flight and 1.2 for gliding). Although sequences were selected based on steady flight, this suggests that either the birds were accelerating during measurement, that our method results in an overestimate, or a combination of both. The method is well-established and has been applied to animal flight in previous studies where it has appeared to perform well - i.e. the mean lift estimate has been close to weight support, although as with most behavioural studies, there is considerable variation (for example, a study on hawkmoths where *L/W* = 1.02±0.49 [8]).

Second moment of area is known to correlate with lift and third moment of area with profile power ([18]–[19]). The third moment of area of the wings was lower for the flapping bird than for the gliding bird (Table 1), so we can rule out the possibility that the increase in profile drag in the flapping bird is due to wing shape alone because the change is in the opposite direction. This indicates that the profile drag we have inferred gives a conservative estimate of the effect that the flapping motion had on profile drag.

The wings of swifts are long and slender (*AR* = 10.3 and 9.8 for the flapping bird and the gliding bird in this study, respectively), tapering towards the tips and with a very short arm-section (section between shoulder joint and wrist joint) the hand section therefore makes up the majority of the total wingspan ([9], [13], [15], [22]). This means that, for a swift, the wrist is located far inboard on the wing, presumably allowing only little active control of spanwise local angle of attack and camber at the more distal parts of the wing. The most effective way to account for the spanwise velocity gradient is to twist the wing such that the local angle of incidence (the angle the wing makes towards the horizontal plane) is reduced towards the tip, keeping the angle of attack (the angle the wing makes towards the oncoming flow) constant. This has been shown to be the case for desert locusts [23], which elegantly compensate almost perfectly for this velocity gradient with spanwise twist in the broad hindwing. This twist is adapted for flapping flight and the locust have no active control of the shape of the distal parts of the wing. Since we can assume that swifts are similarly restricted in their control of the distal parts of the wing, they will not be able to adjust their morphology to both the flapping motion and to the gliding configuration. The results here suggest that the swift wing shape is more tuned towards efficient flapping flight rather than prioritising efficient gliding with respect to the generation of lift, both in terms of the wing twist and the tapering planform.

The wake generated by the tail of the swift in gliding flight has a detrimental effect on the downwash distribution and consequently the span efficiency (Fig. 2). Although Figure 2 shows the most severe case for the sake of clarity, the swift did use the tail to some extent either to increase lift or to stabilize its flight in most of the recorded gliding sequences. One might argue that this is an artefact of the wind tunnel environment since the bird is restricted to a confined volume making it more likely that it has to manoeuver constantly to stay in place. This cannot be excluded entirely but, firstly, the birds were able to fly steadily in the tunnel (as evidenced by prolonged periods within the measurement area) and, secondly, the conditions were the same for the gliding and flapping flight sequences. Hence, the relative performances are still valid, even if a swift in free flight in its natural environment could perform better than a swift in the wind tunnel. That notwithstanding, when the effect of the tail wake was removed, average span efficiency in gliding was increased to 0.47 (from 0.42 when including the tail wake) which is still well below the average span efficiency of 0.62 in flapping flight, and indeed still beneath the lowest of our measurements during flapping flight (*e*_i_ = 0.59). Thus, the majority of the difference in performance between the two flight modes is attributable to the combination of wing shape and kinematics.

## Concluding Remarks

We found that, despite the common swifts being renowned for their gliding behaviour, they performed better in terms of efficiency of lift production in flapping flight than in gliding flight in our wind tunnel experiments. Swifts use both of these flight modes in their natural flight but, because of their unusual wing design with short arm section and long hand section, they cannot adapt their wings to fully suit both the requirement for gliding and flapping. Our results suggest that swift wings are primarily adapted to efficient flapping flight, minimising their costs due to induced drag when other energetic requirements are at their highest.
